# Optical Microfiber Technology for Current, Temperature, Acceleration, Acoustic, Humidity and Ultraviolet Light Sensing

**DOI:** 10.3390/s18010072

**Published:** 2017-12-28

**Authors:** George Y. Chen, David G. Lancaster, Tanya M. Monro

**Affiliations:** Laser Physics and Photonic Devices Laboratories, School of Engineering, University of South Australia, Mawson Lakes, SA 5095, Australia; david.lancaster@unisa.edu.au (D.G.L.); tanya.monro@unisa.edu.au (T.M.M.)

**Keywords:** optical, microfiber, fiber, taper, coil, resonator, sensor, current, temperature, acceleration, acoustic, ultraviolet, humidity

## Abstract

Optical microfibers possess excellent optical and mechanical properties that have been exploited for sensing. We highlight the authors’ recent work in the areas of current, temperature, acceleration, acoustic, humidity and ultraviolet-light sensing based on this exquisite technology, and the advantages and challenges of using optical microfibers are discussed.

## 1. Introduction

The technology and applications of optical fibers have advanced rapidly in recent years. Fiber-optic sensors are commercially successful and well established in industries including healthcare, environment, smart buildings, factories, transportation, retail, security and defense. Being light-driven, light read-out, and requiring no wires provides advantages over their traditional electrical counterparts, which can include higher sensitivity, wider detection-bandwidth, higher-temperature operation, electromagnetic immunity, inertia in combustive environments, robustness and an inherent distributed sensing capability [1]. The physical dimensions and the minimum bend radius of an optical fiber sets a lower limit on the final package size of bent- or coiled-fiber-based sensors.

Optical microfibers (OMs) with their bend insensitivity extend the established capabilities of fiber-optic sensors and allows fiber-optic sensors to be applied where working space is stringent or physical intrusion must be minimized, while maintaining the same level of performance despite miniaturization. Although small-core microstructured fibers [2] can achieve comparable bend loss to OMs when the diameter and numerical aperture of their cores are matched, the bulkier outer-cladding of exposed-core fibers (ECFs) induces greater compression/tensile stresses that could possibly break first when decreasing the bend radius [3]. The emergence of OMs [4,5,6] has opened up a wealth of scientific innovations. OMs have the potential to address emerging sensing challenges with its range of enabling properties, namely larger evanescent field, stronger mode confinement, bend insensitivity, lower stiffness and higher configurability. The latter two attributes are unique to OMs. 

In this article, recent advances by the authors in the areas of current, temperature, acceleration, acoustic, humidity, and ultraviolet-light sensing are highlighted and discussed. For current sensing, ways to reduce the size of the sensor head as well as techniques to overcome the detrimental effects of linear birefringence for higher sensitivity are presented. For temperature sensing, mobility is introduced to elevate the practicality of existing OM-based sensors. For acceleration sensing, OMs are combined with a performance-proven flexural-disc design to demonstrate potentially ultra-sensitive accelerometers. For acoustic sensing, OMs were used for the first time for measuring acoustic signals, in conjunction with a well-established air-backed mandrel design for high sensitivity. For humidity sensing, the fastest-ever hygrometer is demonstrated by combining OMs with thin polyelectrolyte coatings. Lastly, for ultraviolet-light sensing, a new concept sensor is envisaged that aims for experimental validation in the near future.

## 2. Fabrication

**Figure 1:**
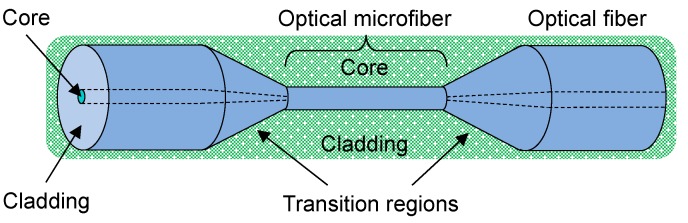
Schematic diagram of an optical microfiber. An example of the new cladding is a liquid analyte (green background).

Optical fibers are commonly made of fused silica, with dopants allowing precise tailoring of refractive index (RI). A typical OM shown in Figure 1 is the uniform waist of a biconical optical fiber taper, with a diameter similar to the wavelength of light in free space. Light is guided along the core of the standard optical fiber until the taper transition. Then, the original core diffuses and the cladding becomes the new core, with the external medium being the new cladding. At the second taper transition, the guidance reverts back to the original conditions.

**Figure 2:**
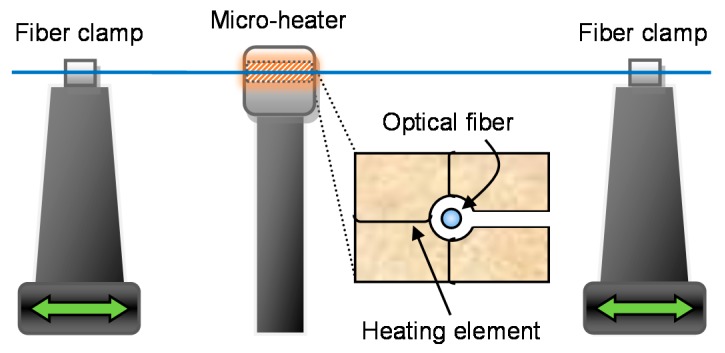
Schematic diagram of the modified flame-brushing technique.

Although there are many fabrication methods [7], OMs are typically produced by heating and stretching regular-sized optical fibers. The commonly used modified flame-brushing technique (MFBT) is shown in Figure 2. The result is a biconical taper that provides a smooth, low-loss connection to other fiberized components. By controlling the pull rate during the fabrication process, the taper diameter profile can be tailored to suit the application [8]. The RI, nonlinearity, optical transparency, absorption, and phonon energy amongst other properties cannot be changed by tapering alone.

By using alternate materials to manufacture OMs [9], properties such as RI, nonlinearity, optical transparency, dopant concentrations, and phonon energy can be tailored. Glasses used include phosphate, tellurite, lead silicate, bismuthate, chalcogenide, while polymers can introduce new functionalities such as enhanced porosity.

By combining a wide range of materials with the optical and mechanical qualities of OMs, a broad palette of design options are available for researchers to develop new types of transducers for sensing applications [10]. OMs are useful for sensors because they allow light to be pulled out of the core to interact with the surroundings, while retaining strong mode-guidance.

**Figure 3:**
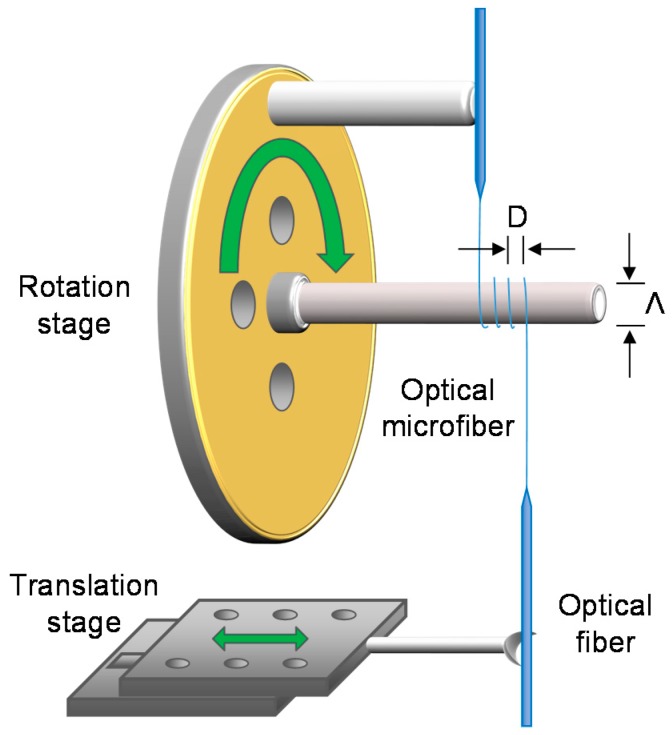
Schematic diagram of a coiling rig setup.

OMs can be manipulated into coils, loops, knots and other geometries [11,12,13] to increase sensitivity (e.g., alignment of light propagation direction with magnetic field) and reduce physical size (e.g., denser packing). For example, microfiber coil resonators (MCRs) involve coiling an OM onto itself to allow evanescent overlap and coupling of the guided mode in the adjacent turns. These micro-resonators can attain high Q-factors [13] that enhance the optical response to a measurand via the recirculation of light within a compact cavity. Figure 3 shows a coiling rig to perform the coiling process after the OM is fabricated, before the MCR is embedded in polymer resin which acts as robust packaging.

## 3. Optical and Mechanical Properties

### 3.1. Optical Confinement and Evanescent Field

**Figure 4:**
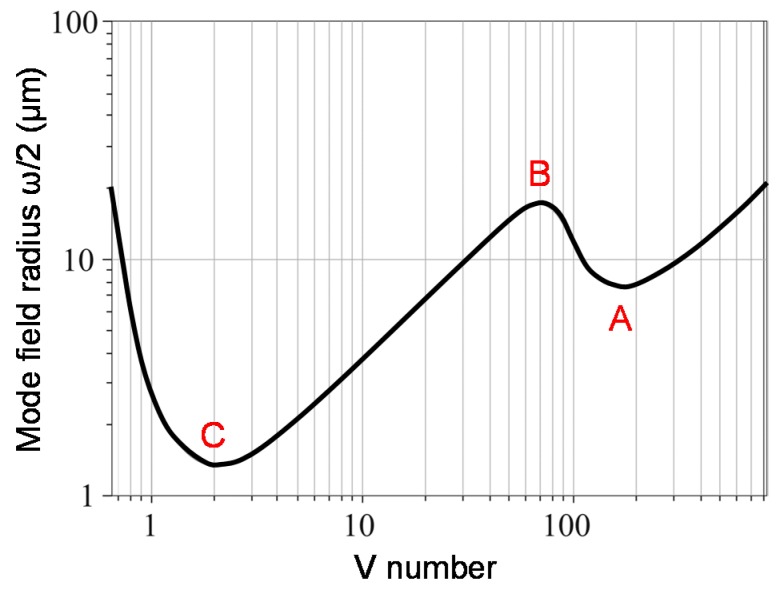
Dependence of the mode-field radius on the cladding V-number of a regular-sized optical fiber during the tapering process [5].

Mode confinement during mode guidance can allow low-loss propagation inside the core of the OM, which can be expressed in terms of the mode-field diameter (ω) that is a function of the V-number [5]. By tapering a regular-sized optical fiber according to Figure 4, the confinement drastically changes. Initially, the light is guided by the core-cladding interface. When the OM radius (*r*) starts to decrease, the V-number decreases and the light becomes more tightly confined within the core, until ω drops to a local minimum point (A). For smaller V-numbers, the light leaks into the cladding. The new guidance by the cladding-surrounding interface causes *ω* to hit a local maximum (B). By further reducing the V-number, the light is confined tighter within the cladding, until *ω* reaches the minimum point (i.e., single mode) at V = ~2 (C). The region below V < 2 is typical of OMs, where *ω* can be much greater than r, and a large portion of the total power resides within the cladding. For V < 0.6, *ω* can continue to expand until it becomes orders of magnitude larger than *r*.

**Figure 5:**
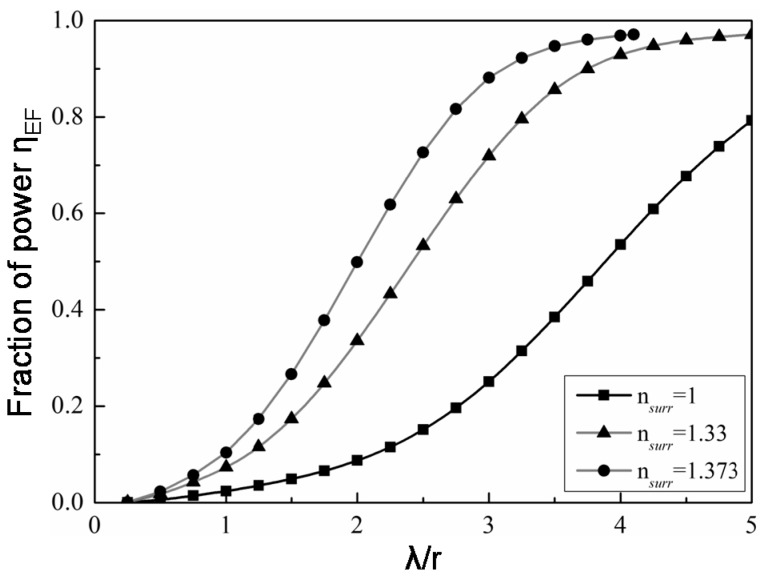
Dependence of the power fraction propagating in the evanescent field on the wavelength-to-radius ratio in different refractive index surroundings [5].

When V << 1 for a tapered regular-sized optical fiber, a large portion of the total power resides in the evanescent field within the cladding [5]. The extension of the evanescent field and the fraction of power (*η_EF_*) propagating in it depend on the *λ*/*r* ratio. Figure 5 shows the nonlinear dependence of *η_EF_* on the *λ*/*r* ratio for silica OMs (i.e., core RI of *n* = 1.444) with different surrounding RI. When the surrounding medium is air, *η_EF_* reaches 0.5 at *λ*/*r* = 4, meaning that half of the power is propagating outside the OM when its radius is a quarter of the wavelength of light. *η_EF_* increases with increasing surrounding RI for the same *λ*/*r* ratio. A large evanescent field is particularly important in resonators, where high Q-factors rely on self or inter-turn coupling. To enhance the evanescent field, low-loss polymers can be used to embed (i.e., submerse) the OM.

### 3.2. Propagation and Bend Losses

The greatest contributions to loss come from surface roughness, diameter non-uniformity, and impurities associated with the OM and its surrounding medium [14]. The attenuation increases for decreasing OM diameter. This can be explained by the stronger interaction between the field intensity of the light and the surface of thinner OMs. A theory of non-adiabatic intermodal transitions was developed to investigate what is the smallest OM that can still transmit light [15]. The guided mode was found to vanish at a threshold value of what is approximately one order of magnitude smaller than the wavelength of light. OMs have relatively low bend-losses, mainly due to the large RI contrast between silica and air. For example, a bend loss of <1 dB was measured for a 90° turn with a 5 µm bend radius of a 530 nm diameter air-clad silica OM [16]. This gives rise to highly compact devices with complex geometry.

### 3.3. Mechanical Strength

Although OMs have very small dimensions, they possess an extraordinarily high strength, due to the smaller flaw size of surface imperfections. The tensile ultimate strength of OMs fabricated by the MFBT was found to be much higher than those made by the self-modulated taper-drawing technique (SMTDT) [17]. SMTDT starts with a broken OM made by the MFBT. Further tapering with a hot sapphire rod is self-modulated in terms of decreasing tensile force as the center of bending shifts from the thicker end of the taper to the thinner end. This results in smaller forces for drawing thinner wires, which helps to keep the taper from breaking under unpredictable drawing conditions.

## 4. Current Sensing

This section recounts the chronological development of current sensors and independent current-sensing techniques using OMs and the Faraday effect. To begin with, the compactness and gigahertz detection bandwidth capability of OM-based current sensors are introduced. Then, the resonance ability of the sensor head is presented and demonstrated for sensitivity enhancement. To address the power-stability issue of resonators, techniques based on RI chirping (i.e., passive) and piezo-electric tuning (i.e., active) are discussed. To further refine the sensor head, the problem of birefringence is considered by first critically appreciating the previous contributions relating to eliminating bend- and packaging-induced birefringence, before presenting a solution in the form of the spun optical microfiber (SOM). The fabrication details and characterization results are followed by current sensing trials. Moreover, the means to achieve efficient Faraday rotation in both birefringent and non-birefringent microfiber loop resonators (MLRs) is theoretically analyzed. Lastly, a conceptual post-fabrication technique is analyzed as an alternative to SOM for countering the birefringence-induced reduction in the sensitivity of microfiber coil (MC)-based current sensors.

### 4.1. Wide Detection-Bandwidth

**Figure 6:**
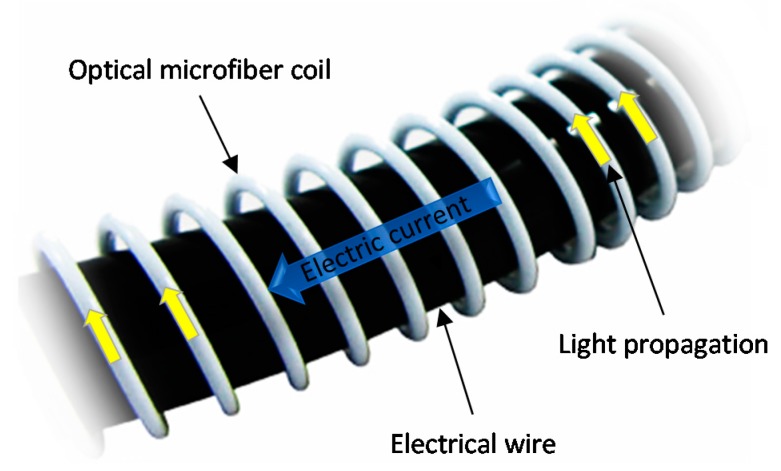
Schematic diagram of the microfiber-coil sensor head.

Current sensors are widely used to detect transient electrical faults and partial discharges on DC lines, in order to protect high power equipment and components. Fiber-optic current sensors that exploit the Faraday effect [18] have attracted interest due to their resilience to electromagnetic interference (EMI) and wide dynamic range. The simplest alternative sensing mechanism is thermal effects, which has been demonstrated with a microfiber knot resonator by Guo et al. [19], a MCR by Belal et al. [20], bend inline OM interferometer by Jasim et al. [21], and graphene-oxide-MC by Yan et al. [22]. They have the benefit of simplicity, being resonance and interferometry based, but are unstable and lack the dynamic range, detection bandwidth and response time achievable with the Faraday effect. Other sensing mechanisms include magneto-optic fluid by Li et al. [23], which is temperature insensitive but can be affected by stray magnetic fields. In addition, strain-based current sensing by Yan et al. [24], which is simple but temperature dependent. The Faraday effect describes a current-induced magnetic field aligned parallel to the direction of light propagation, inducing a rotation in the polarization of light. Optical fibers can allow the direction of light propagation to be aligned with that the magnetic field. A popular design consists of an optical fiber coiled around a straight current-carrying wire rather than a conducting wire wound around a straight optical fiber, as shown in Figure 6. Not only is this arrangement more viable in a real measurement environment, it also minimizes the load impedance and thermal effects along the current-carrying wire. As a result, the frequency of alternating current (AC) signals can exceed hundreds of kilohertz that is typical of the coiled wire arrangement. Moreover, it has lower cross-sensitivities than those of the straight-fiber configuration, which are ideal for the measurement of 1-dimensional magnetic signals in 3-dimensional space. Conventional fiber-optic current sensors are relatively bulky [25,26], because of the large bend radius required to maintain sufficiently low optical-loss. Moreover, the relatively low Verdet constant of silica necessitates a large number of fiber turns to produce a measurable Faraday rotation. Owing to their long optical path lengths (OPLs), the transit times of light (i.e., integration times) are long, and as a result their detection bandwidths cannot exceed tens of megahertz. Overcoming this hurdle will open the doors to applications in portable electronic devices and defence (e.g., electromagnetic pulse).

The first experiment on OM-based current sensing using the Faraday effect was undertaken by Belal et al. [27]. The bend insensitivity of OMs enabled the size of the sensing fiber coil to be down-scaled (i.e., the same number of turns and a shorter OPL, instead of a larger number of turns and the same OPL). The short OPL minimized the transit duration of light through the MC, and thus boosted the detection bandwidth. Consequently, it allowed for the interrogation of very-high frequency currents, potentially of the order of gigahertz. This was something never before possible with regular-sized optical fibers. In the experimental demonstration, a 0.5 mm diameter copper wire tightly coiled with 25 turns of 5 μm diameter OM only required an OM length of ~10 cm. The maximum frequency bandwidth tested was 400 kHz from a pulsed current source, which is far from its theoretical limit of the order of gigahertz. The sensitivity is 16.8 ± 0.1 µrad/A.

Following the work of Belal et al. [27], further tests by Chen et al. [28] confirmed the ultra-fast response times and wide detection-bandwidths of the MC based current sensor. Although a 5 μm diameter OM of 30 mm length is theoretically capable of sensing currents in the gigahertz regime, full-width-half-maximum pulse durations of ~7 ns and frequency bandwidth of ~57 MHz have been detected by using a similar polarimeter setup, which was limited by the modulation bandwidth of the available current pulse generator. It employed 10.5 turns of OM around a copper wire of 1 mm diameter. The MC and the wire were packaged by embedding in UV-curable polymer (Luvantix SSC, Warren, NJ, USA, Efiron PC-373 AP) for robustness.

Chen et al. [29] have also shown that due to the current integration effect of the MC, the Faraday effect cancellation and pulse broadening grow with increasing signal frequency, which result in suppression and distortion of the optical response. At integer multiples of a cycle period, the net rotation is always zero due to the total cancellation of the opposite signs of Faraday rotation. The detection bandwidth is nominally derived near the –3 dB level of response, where its half-cycle period is equal to or more than the transit duration (Δ*T*) of photons through the MC. For regular-sized optical fibers hosting co-propagating electrical signal and light, Δ*T* is the difference in the transit duration between light traveling through the sensing fiber coil (Δ*T_coil_*) and the magnetic-field signal along the electrical wire (Δ*T_wire_*). For the counter-propagating case, the figure would show that Δ*T* is the sum of Δ*T_coil_* and Δ*T_wire_*. This is because there is a positive (i.e., co-propagating) or negative (i.e., counter-propagating) displacement of Δ*T_wire_* between the photons at the input and output of the sensing fiber coil, within the default integration time of Δ*T_coil_*. For OMs with small diameters and/or electrical wires with large diameters such that Δ*T_wire_* is less than a tenth of Δ*T_coil_*, the former can be approximated as zero. The expression for detection bandwidth is a function of the effective RI seen by the guided mode (*n_eff_*), the length of the uncoiled optical fiber (*L_fiber_*), the speed of light in free space (*c*), the length of the enclosed wire (*L_wire_*), and the propagation speed of the electrical signal (*c*′):

(1) $$B_{w}=\frac{1}{2\times \Delta T}=\frac{1}{2\times \left(\Delta T_{coil}\mp \Delta T_{wire}\right)}=\frac{1}{2\times \left(\frac{n_{eff}L_{fiber}}{c}\mp \frac{L_{wire}}{c^{\prime }}\right)}$$

To increase the detection bandwidth, the transit duration must be shortened through reducing the length of the uncoiled OM. The sensitivity can be increased by using a larger number of OM turns, with the limiting factor being the maximum length of OM that can be fabricated. Since there is a conflicting requirement of OPL, there is a trade-off between sensitivity and detection bandwidth. Optimization of the MC parameter will deliver a good balance of the sensor parameters. To increase the rotation sensitivity, the AC component of the detected differential signal must be divided by the direct current (DC) component or total power in real-time, which eliminates laser intensity noise. The ultimate compactness of the sensor head is restricted by the diameter of the copper wire to be measured, followed by the minimum bend radius of the OM. MCs are not the ideal candidates for measuring large-diameter wires/cables, because the advantages of small dimensions and wide detection-bandwidth are lost. To make the sensor head more practical, a sliding tube mechanism must be used instead of permanently binding the MC to a single electrical wire, similar to the design of the temperature sensor in Section 5. The non-contact approach will allow the same sensor head to probe a number of wires instead of being single-use. It will also avoid loading the system with additional impedance that restricts the current flow. Since this work, other research groups such as Zhao et al. [30] and Talataisong et al. [31] have explored alternative ways to fabricate current/magnetic-field sensors based on the Faraday effect.

### 4.2. Resonantly Enhanced Sensing

MC-based current sensors demonstrate exceptional compactness and wide detection-bandwidths. However, the sensitivity still needs to be improved. To increase the sensitivity, one approach is to employ a significantly longer OM. However, this is not feasible with most conventional tapering rigs. Overcoming this hurdle can facilitate the development of compact current sensors with sensitivities competitive to existing counterparts made with regular-sized optical fibers.

**Figure 7:**
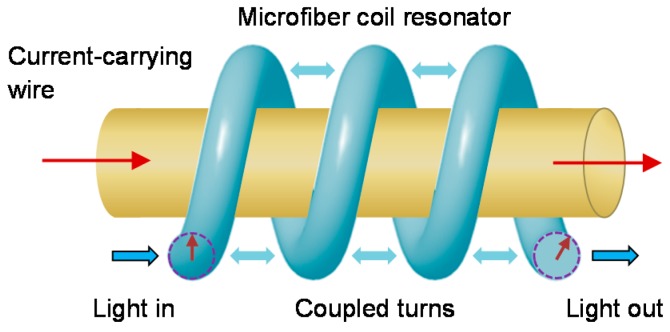
Schematic diagram of the microfiber coil resonator sensor head being interrogated by current. The directions of light propagation and mode coupling are shown as arrows. The arrows in dashed-circles represent the Faraday rotation.

To address the problem of OM length limitation, MCRs were explored by Chen et al. [32]. The advantage of MCRs is that they potentially only require a few turns as their sensitivity depends more on the proximity from critical coupling and the detuning from resonance than the physical OM length. Resonantly enhanced Faraday rotation, shown in Figure 7, can arise due to the larger power transfer at the resonant wavelength. Furthermore, the balance between sensitivity and detection bandwidth can be tuned via the wavelength of the input light. Despite the issue of stability, MCR-based sensor heads can offer significantly wider detection-bandwidth compared to the regular-sized fiber-coil sensor heads of conventional fiber-optic current sensors.

An experimental demonstration was carried out using a 3-turn MCR of 2 μm core diameter and 10 mm uncoiled length around a 0.5 mm diameter copper wire. The sensor head and wire were packaged in UV-curable polymer. The sensitivity was observed to be 4.4 ± 0.6 μrad/A for a current signal of 30 Hz at the resonant wavelength. An enhancement factor of 3.1 was observed from the comparison between the sensitivity at a resonant wavelength and that at an off-resonance wavelength [32]. This indicates that resonantly enhanced Faraday rotation is possible, and thus MCRs can potentially match the sensitivities of MCs with far shorter lengths of OM. Compared to the earlier work by Belal et al. [27], where a sensitivity of 16.8 µrad/A was reported, the sensitivity of the MCR is lower. The detection limit is 931.8 mA, from dividing the rotation detection-limit (4.1 μrad) by the sensitivity (4.4 μrad/A) at the resonant wavelength.

To increase the enhancement factor and thus raise the sensitivity, it is imperative to fabricate MCRs of higher Q-factor and lower linear-birefringence. However, there exists a fundamental limit to the linear birefringence determined by the coupling coefficient. Therefore, further improvement relies on new fabrication techniques. The rotation resolution can be increased by suppressing polarization-dependent loss, which allows the MCR to operate at a quadrature point for high signal-to-noise ratio. To increase the detection bandwidth, the transit duration must be shortened through decreasing the OM turn length and reducing the Q-factor (i.e., shortening the effective roundtrip time of light). The Q-factor can be increased by lowering the attenuation through removing air bubbles from the polymer packaging.

### 4.3. Passive and Active Resonator Stabilization

Since the transmission spectrum of a MCR is governed by its geometry, even minor deviations in the position of the OMs can significantly change its resonance characteristics and induce a significant resonance detuning. Temperature drifts are known to produce a spectral shift of ~100 pm/°C with silica MCRs embedded in polymer PC-373 [33], and ~10 pm/°C when embedded in Teflon resin [34]. These changes are due to a combination of thermal-optic, thermal expansion and stress-optic contributions. Teflon can be used achieve high-temperature operation >300 °C [34]. Achieving temperature insensitivity will make such resonators viable for not just current sensing, but also for other applications. The hurdle to overcome is to optimize the design of the sensor head such that the combined thermal effects of the various materials cancel out, or select materials with low thermal-expansion and thermos-optic coefficients both the OM and the packaging.

**Figure 8:**
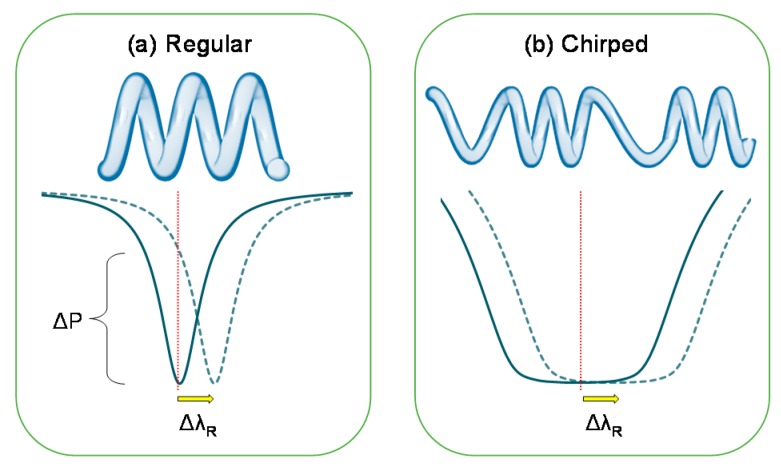
Effect of ambient temperature-induced resonance shift on the transmission spectrum of a: (**a**) 3-turn microfiber coil resonator, and (**b**) 12-turn chirped microfiber coil resonator. The dashed lines facilitate a comparison of the output power before and after the resonant wavelength shift.

In the work of Chen et al. [35], the chirped MCR is presented and its ability to keep the MCR on resonance despite temperature drifts are discussed. For resonantly enhanced sensors and devices exploiting the recirculation of light at the resonant wavelength, a wider margin at zero detuning can considerably improve the power stability of the resonator output in the presence of thermal fluctuations (i.e., <10 Hz) as well as seismic and acoustic vibrations (i.e., <1 kHz). As depicted in the transmission spectra of Figure 8a, a small temperature-induced resonant wavelength shift translates into a large optical power modulation for a standard MCR. However, by chirping the MCR geometry in terms of: (a) periodically decoupling regions to form cascaded MCRs; and (b) modifying the resonant wavelength of each new MCR; the resonant condition can be widened such that the output power of the resonator is constant across a broader wavelength range. Figure 8b shows a chirped MCR where the reduction in transmission is negligible, such that the initial resonant wavelength remains on-resonance.

The chirping of the MCR could possibly be realized by using femtosecond-laser inscription to modify the RI of local areas of polymer surrounding each pair of turns, such that the OPL and thus resonant wavelength is changed accordingly. The geometrical arrangement in Figure 8b can be achieved by coiling the paired turns with a smaller winding angle and a larger winding angle for the uncoupled regions. The paired turn configuration also provides higher feasibility for a femtosecond-laser inscription, since high spatial resolution is not required when operating on a larger surface area. The reproducibility of such structures could be very high with high-precision translation stages.

**Figure 9:**
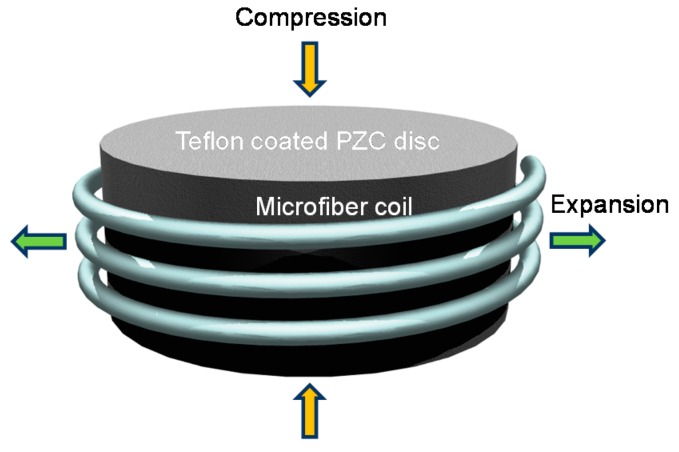
Schematic diagram of the microfiber coil resonator attached to the Teflon-coated piezo-electric ceramic disc.

Unlike the previous technique that relies on passive stabilization, the complementary technique [35] is based on active stabilization of the resonant wavelength by reshaping the MCR, and thus shifting the transmission spectrum to compensate for any temperature drifts. This approach offers the advantage of continuous operation, but requires inserting an electrically active element into the MCR package. Owing to the electro-mechanical effect of the piezo-electric ceramic (PZC) disc illustrated by Figure 9, strain-induced changes in the OPL of the bound OM modifies the phase condition of the MCR for resonance, and thus creates a shift in the resonant wavelength. For negative voltages, the disc waist expands such that each turn of the MCR increases in OPL despite a small offset by the stress-optic effect, and thus the resonant wavelength undergoes a positive shift.

An experimental demonstration was conducted using a 2.5-turn MCR of 2 μm core diameter and 25 mm uncoiled length, wrapped around a PZC disc of 3 mm diameter and 1 mm thickness. The relationship between the applied voltage and the measured resonant wavelength shift was observed to be approximately linear for the tested range. A responsivity of 67.5 ± 8.0 fm/V was measured.

To increase the tunability of the disc, a longer OM and longer wavelength must be used. A disc of higher piezo-electric strain coefficient would also be beneficial. The power stability of the sensor head could be improved further by combining the passive and active stabilization techniques, such that a PZC disc is integrated inside a chirped MCR. The foreseeable challenge would be to modulate all the OM turns in such a way that the collection of resonances shift by the same magnitude in the wavelength domain, in order to prevent distortions in the combined spectral shape.

### 4.4. Spun Optical Microfiber (SOM)

Although the minimum permissible bend radii of OMs are significantly smaller than those based on regular-sized optical fibers, the same problem of linear birefringence arises after packaging MCs in UV-curable polymer. For MCRs, there is always a fundamental contribution in addition to the environmental contribution, in which linear birefringence arise from the polarization-dependent coupling coefficients. In the presence of linear birefringence (i.e., non-zero differential phase), the net power transfer between the fast- and slow-axes due to Faraday rotation is maximum after the first quarter of the polarization beat length (PBL). However, it reduces to zero over a half of the PBL, before building up again in a cyclic pattern. The initial differential phase bias can be set to π/2 or 3π/2, plus integer multiples of 2π, in order to extend the maximum usable fiber length to half a PBL. Addressing this issue will enable up-scaling of fiber length to achieve high sensitivity. Numerous solutions have been reported over the years [36,37,38]. The spun optical fiber pioneered by Laming et al. [38] was introduced as an evolutionary step that has sufficient resistance to external effects without compromising the Faraday effect. A more successful variant of the spun fiber is based on highly linearly birefringent preforms [38]. It is made by spinning the preform during the drawing process to impart a rapid built-in rotation of the fast and slow axes. The resulting optical fiber becomes elliptically birefringent. By carefully choosing the spin rate relative to the intrinsic linear birefringence, the resulting optical fiber can be considered as a compromise between an isotropic waveguide (i.e., weak linear birefringence but strong circular birefringence) that it has a high responsivity to magnetic fields and a low sensitivity to temperature and wavelength drifts, and a highly linearly birefringent fiber (i.e., strong linear birefringence but weak circular birefringence) that has a high resistance to external perturbations. The challenge is to apply existing techniques to fragile OMs without causing undesirable damage or deformation.

**Figure 10:**
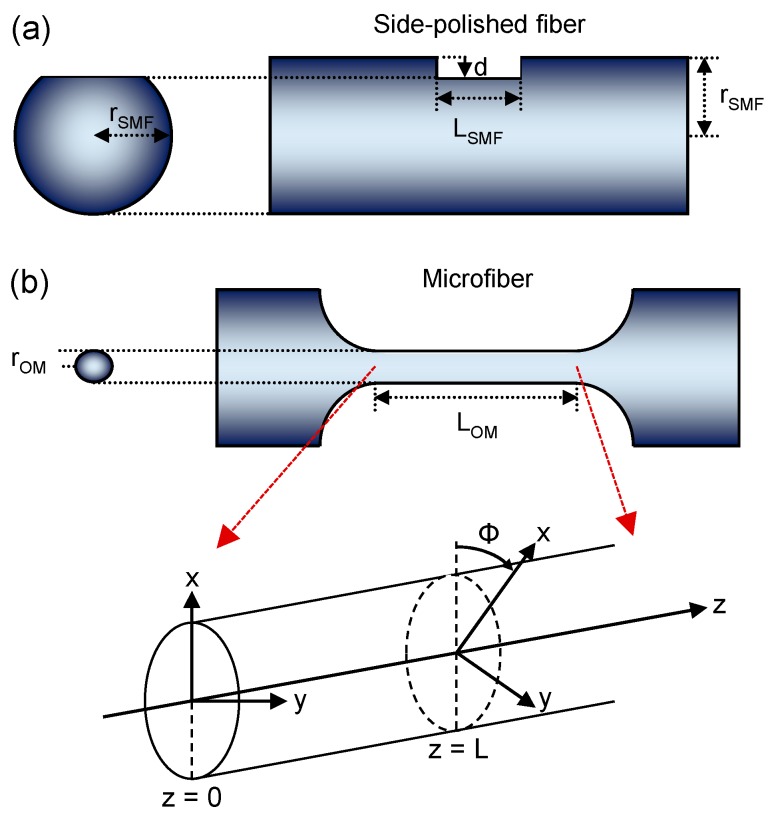
Side view of (**a**) a side-polished fiber, and (**b**) the waist of a tapered fiber. Expanded: Schematic diagram of the spun microfiber with a constant spin rate Ф along fiber axis *z*. Typically *r_SMF_* = 62.5 μm, *r_OM_* is between 1 μm and 10 μm, *L* is between 0.1 μm and 1 m, and Ф is between 0 and 100 π/cm.

Owing to the effectiveness of the spun fiber technique and the feasibility for the OM to be spun in a similar fashion, it was of interest to study the SOM and its potential for current sensing. Chen et al. [39] presented the first fabrication of SOM and the anticipated improvements to sensitivity were experimentally confirmed. The fabrication process illustrated in Figure 10 involves tapering while rotating a highly birefringent optical fiber made by side-polishing. The different optical confinement along different fiber axes introduces local linear-birefringence that counters external effects, while the rotation of its axes keeps detrimental effects related to differential phase and Faraday rotation to a minimum.

To demonstrate that MC-based current sensors based on SOM are more resilient to bend- and packaging-induced linear birefringence than those made using standard OM, three MC samples were fabricated based on OM and another three for SOM. The six samples were all 2 μm diameter and 30 mm length with semi-adiabatic taper transitions of *ψ* = 0.3 [40]. A spin rate of Ф = 24 π/cm (see Figure 10) was chosen for the SOM samples, with the intrinsic linear birefringence being ~1 × 10^−3^. A thin layer of UV-curable polymer was deposited on a 1 mm diameter copper wire before and after coiling the optical fiber. Each MC had 7.5 turns with a large winding pitch (e.g., >0.5 mm) to prevent mode coupling between adjacent turns. Lastly, each sample was UV-cured. The measured data revealed that SOM-based MC sensor heads were able to reach an average sensitivity of ~8.6 μrad/A. In comparison, the standard OM-based MC samples show less consistency with an average sensitivity of ~5.7 μrad/A. This indicates that spinning the OM offers better suppression of external linear-birefringence. Moreover, the reproducibility of standard OMs is worse due to the unpredictable build-up of linear birefringence during the coiling and packaging process.

To further increase the sensitivity, the spin rate must be increased and the intrinsic linear birefringence must be decreased. The resilience against external perturbations can be strengthened by decreasing the spin rate and the intrinsic linear birefringence must be increased. Since there are conflicting requirements of spin rate and intrinsic linear birefringence, there is a trade-off between sensitivity and susceptibility to external effects. Optimization of the fiber parameters will deliver a good balance of the sensor parameters.

### 4.5. Efficient Faraday Rotation in Birefringent Microfiber Loop Resonators (MLRs)

Resonantly enhanced Faraday rotation was previously demonstrated in MCR-based current sensors. However, the observed enhancement factor was relatively low due to linear birefringence, and the choice of geometry based on the diameter of the host electrical wire. Even if the linear birefringence was suppressed by using SOM, changes in the differential phase due to non-optimized inter-turn coupling can have an unpredictable effect on the sensitivity. Addressing this issue can realize the original motivation of resonance-enhanced OM-based sensor heads, which is to achieve high compactness without loss of sensitivity.

**Figure 11:**
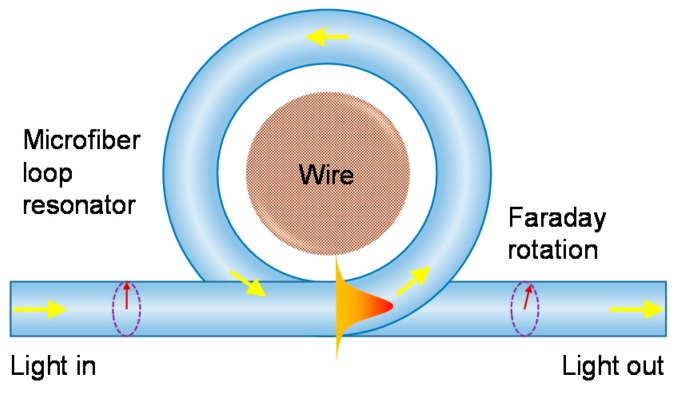
Schematic diagram of a microfiber loop resonator-based sensor for current sensing. The directions of light propagation and mode coupling are shown as arrows. The arrows in dashed-circles represent the Faraday rotation. The horizontal peak illustrates the intensity distribution.

In the work of Chen et al. [41], geometrical optimization was studied for the simpler MLR to overcome the OPL limit. Unlike a typical MCR, a MLR shown in Figure 11 has negligible linear birefringence from its polarization-dependent coupling coefficients due to its much shorter coupling-length. It was shown that efficient Faraday rotation is feasible for sensor heads incorporating birefringent OM, and the maximum Faraday rotation can be obtained for non-birefringent sensor heads. Although efficient Faraday rotation naturally coincides with on-resonance, in the presence of linear birefringence a geometrical relationship is required between the loop length and linear birefringence.

For MLRs with a sufficiently large birefringence-induced resonance separation, efficient resonantly enhanced Faraday rotation can arise when the loop circumference is a quarter of the PBL, and the closed-loop roundtrip phase delay of the eigen-modes in the fast and slow axis are equal to 2π and π/2 respectively for the fast axis on resonance, or 3π/2 and 2π respectively for the slow axis on resonance, all plus an integer multiple of 2π. These phase rules can be converted into geometrical design rules for fabricating MLR sensor heads that can facilitate a large Faraday rotation for multiple closed-loop roundtrips despite the occurrence of linear birefringence. Hence, it would be of interest to experimentally demonstrate this, but it is a challenge to achieve sufficiently high fabrication-precision of the OM lengths. To increase the Faraday efficiency and thus increase the sensitivity, it is possible to set up the MLR with a non-zero initial differential phase.

### 4.6. Post-Processing of Birefringent Microfiber Coils

Optimized MLRs rely on precise geometry tailored to the linear birefringence to ensure that any closed-loop roundtrip of light initializes with a differential phase equal to an integer multiple of 2π, and exits with a differential phase of π/2 plus an integer multiple of 2π. For uncoupled MCs, a methodology of manipulating differential phase is needed such that high sensitivity can be maintained with longer OM lengths. The challenge is determining which positions to modify the OM.

**Figure 12:**
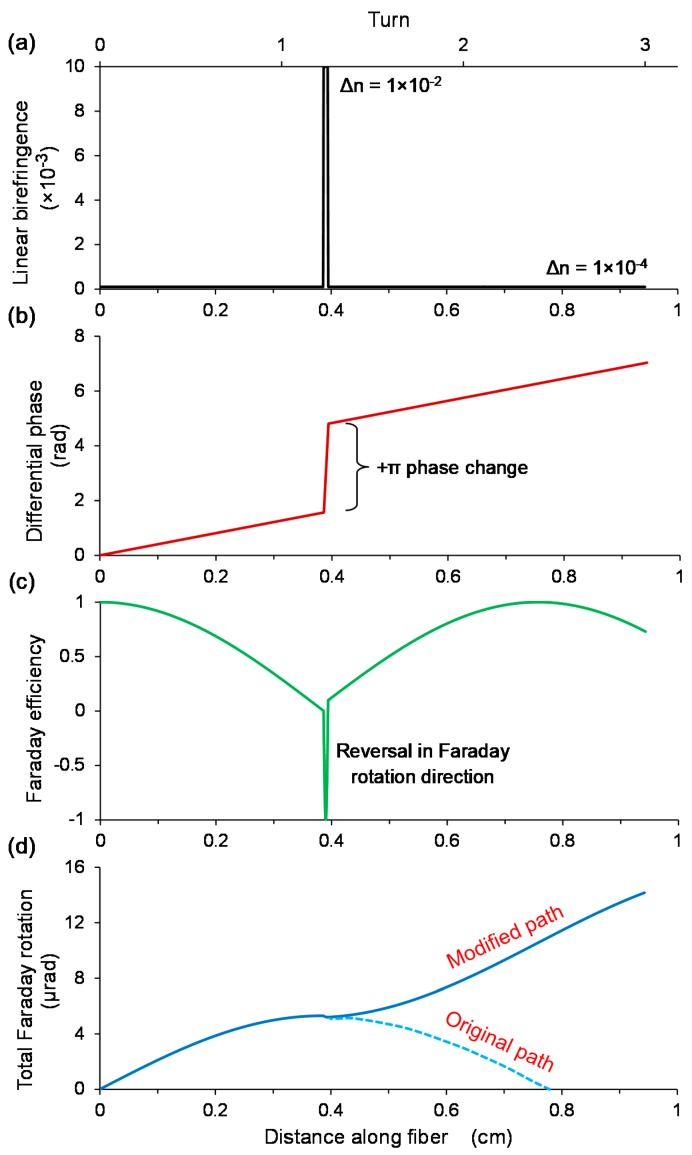
Simulations of birefringence modulation in a microfiber coil to rectify the direction of Faraday rotation for an efficient build-up of sensitivity over fiber length. The impact of changing (**a**) the local birefringence, is reflected in (**b**) the differential phase, (**c**) the Faraday efficiency, and (**d**) the Faraday rotation.

One conceptual approach to this problem was developed by Chen et al. [42], who demonstrated that by manipulating the local birefringence at certain regions along the OM indicated in Figure 12, a gain of ±π in differential phase between the polarized light in the two orthogonal axes of the birefringent OM can prevent the sensor head from entering a state of reversal in Faraday rotation, by jumping to a state that continues with the same direction of Faraday rotation. This approach is predicted to be highly flexible and tolerant to MC fabrication imperfections and post-fabrication linear-birefringence treatment errors. The procedure involves measuring the PBL of a fabricated MC sample, before modifying the local birefringence at initially a quarter of the PBL, then at every interval equaling a half of the PBL. The birefringence change could possibly be performed by femtosecond-laser inscription, though this is yet to be demonstrated. The precision of the localized modification in terms of the fiber position and modification area does not need to be high, as long as the perturbation in differential phase can prolong a unidirectional Faraday rotation. Furthermore, the results of this post-fabrication treatment could be monitored in real-time.

To further increase the sensitivity, the modified sensor head must be used with a Faraday rotator mirror (FRM) to double the OPL and thus Faraday rotation. Since the returning eigen-modes occupy the orthogonal axis, the differential phase evolution is reversed. Combined with the fact that the Faraday rotation is monotonic with the differential phase shift of π and the flip in magnetic field direction, the Faraday efficiency trend for the backward propagation is a mirror image of that of the forward propagation. As a result, the Faraday rotation is doubled regardless of where the FRM is inserted. If a mirror is employed, the fiber length becomes critical in the sense that the fiber length between the final phase-correction point and the mirror must be equal to a quarter of the PBL. This will ensure that the differential phase does not exceed ±π, and thus prevents a reversal in the direction of Faraday rotation before returning to the nearest phase-correction point.

## 5. Temperature Sensing

The common origins of overheating that lead to electrical fires are excessive currents, poor connections and insulation breakdown. A way to determine the necessity of repairs or replacement wires is to measure the temperature distribution along the length of the wire. Defects in the insulation integrity are distinguished by an increase in the heat signature. Electrical thermometers lend themselves to EMI issues, while thermal imaging cameras cannot be used in confined or cluttered spaces. A variety of optical thermometers have been reported in recent years [43,44]. However, most optical thermometers require precise positioning of the sensor head for accurate temperature mapping. MLRs by Zeng et al. [45] and MCRs by Wu et al. [46] have shown sensitivities as high as 280 pm/°C. They have hollow centers suitable for gliding along electrical wires. However, these devices were always immobilized on a flat substrate or cylindrical rod that eliminates their potential advantage. Being able to move the sensor head along the wire makes temperature measurements more practical.

**Figure 13:**
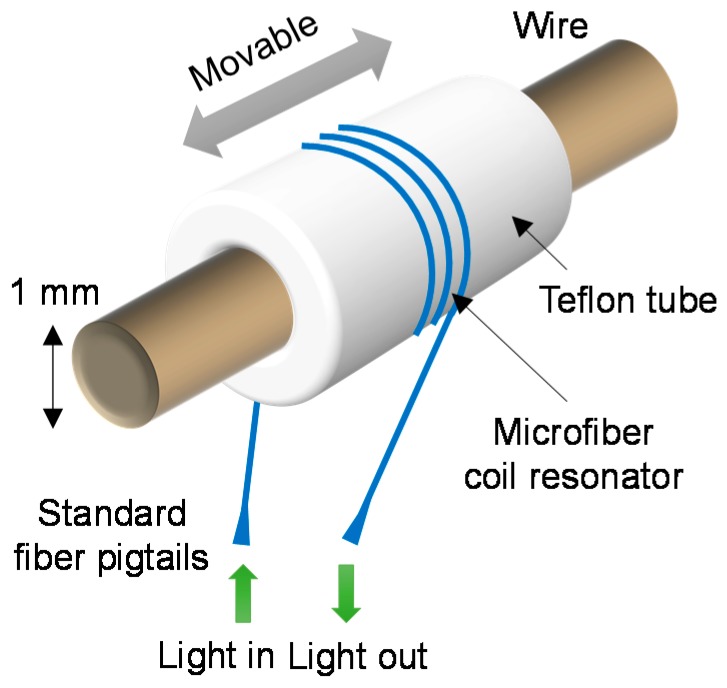
Schematic diagram of the microfiber coil resonator-based thermometer sliding along an electrical wire to measure the local temperature.

To demonstrate viability, a wire-mounted sliding Teflon tube coiled with an MCR was fabricated by Chen et al. [47]. A hollow sliding probe can be used to rapidly inspect long electrical wires for fault location. This is performed by inserting the wire-under-test through the tube, moving the sensor head along the wire, and monitoring the transmission spectrum for temperature-induced shifts. The integrated MCR shown in Figure 13 maps the local temperature to identify positions with insulation faults that can be at risk from electrical arcing. Defects in the insulation integrity are distinguished by an increase in heat dissipation, which changes the OPL and thus the resonant wavelength of the MCR. Additionally, elevation in temperature generated by intense currents can be identified. The ring-shaped detection region facilitates a rotationally symmetric coverage and thus removes the need for accurate positioning of the sensor head, due to a helical configuration of the OM. The minimum bend radii of OMs allow a wide range of wire diameters to be measured, ranging from millimeters to tens of micrometers.

**Figure 14:**
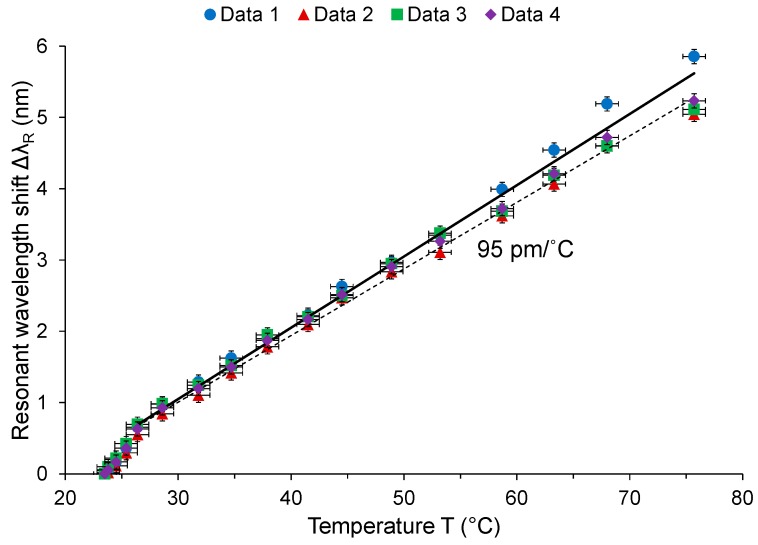
Measured sensitivity with an average linear fit (i.e., dashed line), compared with the simulated sensitivity (i.e., solid line).

A 2.5-turn MCR of 2 μm core diameter and 15 mm uncoiled length was wrapped around a 1.8 mm diameter Teflon tube with a Nichrome wire passing through. The sensitivity was determined as 95 pm/°C between 26 °C and 76 °C, as shown in Figure 14. A detection limit of 0.21 °C was deduced.

To increase the sensitivity, a larger MCR diameter and lower resonance-index (i.e., longer wavelength) must be used. A sliding tube of higher thermal expansion coefficient would also be beneficial. A longer resonant wavelength to track is also preferable. The upper limit of measurable temperature can be extended by replacing the polymer packaging with Teflon, which has a much higher melting temperature in excess of 300 °C. However, Teflon resin is very expensive and large quantities are needed to fully embed the MCR, due to significant shrinkage in volume after drying in air. The ultimate compactness of the sensor head is restricted by the diameter of the Teflon tube, which has to be larger than the range of wire diameters to be measured. It is then followed by the minimum bend radius of the OM.

## 6. Acceleration Sensing

Accelerometers are indispensable tools for measuring displacement/velocity/acceleration in planes, vehicles, vessels, portable electronic devices, earthquake monitoring stations, power plants, space stations and other inertial navigation systems. Fiber-optic accelerometers have several inherent advantages over micro-electromechanical-systems-based accelerometers, including higher sensitivity and better immunity against EMI. Optical accelerometers have been reported in many different forms, with the most common being compliant cylinders/mandrels, demonstrated by Pechstedt et al. [48], and central/edge-supported flexural discs (FDs), presented by Wang et al. [49]. Both of which utilize highly sensitive interferometric techniques. Other types include hollow/multi-core fibers, microloop resonators, weighted reflective diaphragms and FBGs [50]. In-fiber designs are highly compact but are limited by low sensitivity. Although designs based on compliant cylinders/mandrels and weighted reflective diaphragms have some benefits such as very-high sensitivity, they also exhibit narrow detection-bandwidths. FD designs exhibit low sensitivity in small packages, while they tend to have reasonable detection bandwidths up to several kilohertz [51], which is higher than most configurations. Moreover, they offer relatively low cross-sensitivities that are ideal for the measurement of 1-dimensional acceleration signals in 3-dimensional space. However, for portable devices where lightweight and compactness are desirable, FDs face a serious design problem. Addressing this need allows compact accelerometers to be used in aerospace and portable electronics applications.

**Figure 15:**
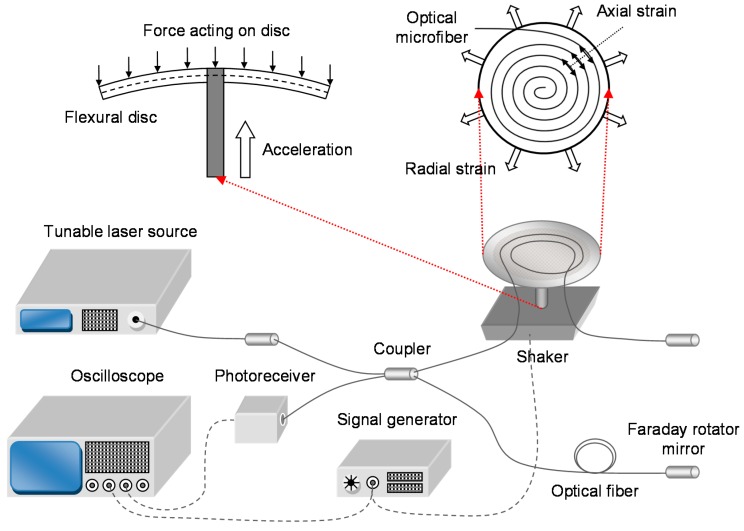
Schematic diagram of the experimental setup with the sensing mechanism illustrated.

Chen et al. [52,53] experimentally demonstrated two OM-based, centrally supported FD accelerometers, as shown in Figure 15. OMs can have bend radii of the order of millimeters without inducing significant optical loss. Therefore, high compactness is feasible without deterioration in expected performance. A smaller disc size also constitutes a higher fundamental frequency and thus a wider detection-bandwidth. Since OMs have remarkably small diameter and low stiffness compared to regular-sized optical fibers, it is predicted that massive lengths can be packed onto the surface of a regular-sized disc (i.e., diameter in excess of several centimeters), leading to a larger response to strain and thus a higher sensitivity. The flexural disc operates by translating vertical movements into flexing angles of the disc, which then deforms the attached OM to produce a change in the OPL and thus phase delay. A Michelson interferometer converts the phase modulation into a power modulation. 

For this single-sided design, the maximum strain-induced phase shift of one OM spiral occurs when the displacement of the accelerometer is comparable to the disc thickness. For larger displacements, the OM can experience compressive strain-induced phase shifts regardless of which side of the disc it is bond to. Therefore, the polarity of its phase shift might not be maintained with increasing displacement if the flexural disc experiences significant bend-induced radial contraction along the neutral surface and insignificant tilt-induced radial extension along the OM plane. This results from: (a) relatively high Young’s modulus; (b) relatively large vertical displacement of the central support; (c) relatively large disc radius, and thus large flexing angle; and/or (d) relatively small disc thickness. For the double-sided design, the maximum strain-induced phase shift of one OM spiral occurs when the displacement of the accelerometer is equal to the central-support height. Hence, the polarity of the phase shift is retained with monotonic vertical displacement.

**Figure 16:**
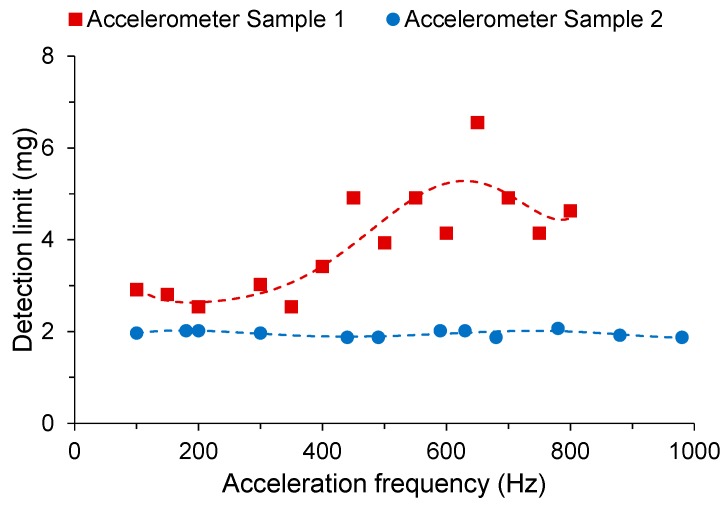
Measured frequency-resolved acceleration detection limits [53], with polynomial fit.

Two different accelerometer samples were compared. Sample 1 used a shorter OM of 10 mm length with a smaller diameter of 2 μm. Sample 2 used a longer OM of 60 mm length with a larger diameter of 10 μm to improve handling during the winding process due to the fragility of OMs. The samples were embedded in low-RI UV-curable polymer on a pyrolytic graphite disc of 25 mm diameter and 0.5 mm thickness. The average sensitivity is 2.0 rad/g for Sample 1, and 4.0 rad/g for Sample 2 at a signal frequency of 500 Hz, as shown in Figure 16. The average detection limits were observed to be 3.9 mg and 2.0 mg respectively. It is to be noted that this is a proof-of-concept demonstration, and to be competitive with commercial accelerometers, both optical and electrical, require the flexural disc to be packed with far longer OM.

To increase the sensitivity, a double-sided design, longer OM and shorter wavelength must be used. The limiting factor is the maximum length of OM that can be fabricated. A disc of larger radius, greater thickness, lower Young’s modulus and higher density will also be beneficial. The phase resolution can be improved by using a Mach-Zehnder interferometer with the balanced-detection scheme to minimize common-mode intensity noise from the tunable laser source. In addition, the Michelson interferometer can be temperature-compensated by attaching the reference arm to the underside of the flexural disc, so that any thermal expansion would equally affect both OPLs. To increase the fundamental frequency and thus increase the detection bandwidth, a disc of smaller radius and greater thickness, with a material of higher Young’s modulus, lower density and higher Poisson ratio is required. Since there are conflicting requirements of disc radius, Young’s modulus and density, there is a trade-off between sensitivity and detection bandwidth. Optimization of the disc parameters will deliver a good balance of the sensor parameters. The ultimate compactness of the sensor head is restricted by the minimum bend radius of the OM.

## 7. Acoustic Sensing

Acoustic sensing is one of the most widely established areas of fiber-optic sensors. Rapid development in this area have been driven by heavy demands from industries including defense. The acoustic signatures of tanks, aircraft, helicopters and submarines can be detected for surveillance and strategic planning. Optical microphones with immunity against EMI based on an air-backed mandrel benefit benefits from their simple yet effective design reported by Wagaard et al. [54], and the potential for high sensitivity compared to a micro-bending OM shown by Xu et al. [55], and tapered interferometers by Chen et al. [56] and Xu et al. [57]. Moreover, it has higher cross-sensitivities than those of diaphragm-based designs, which are favorable for the measurement of 3-dimensional acoustic signals in 3-dimensional space. However, due to the large minimum bend radii of regular-sized optical fibers, very compact designs are not achievable. Moreover, even state-of-the-art bend-insensitive fibers can achieve a bend radius down to several millimeters [58], relatively large fiber diameters result in low sensitivities. Scaling down the size of acoustic sensors is advantageous due to easier and more effective deployment.

**Figure 17:**
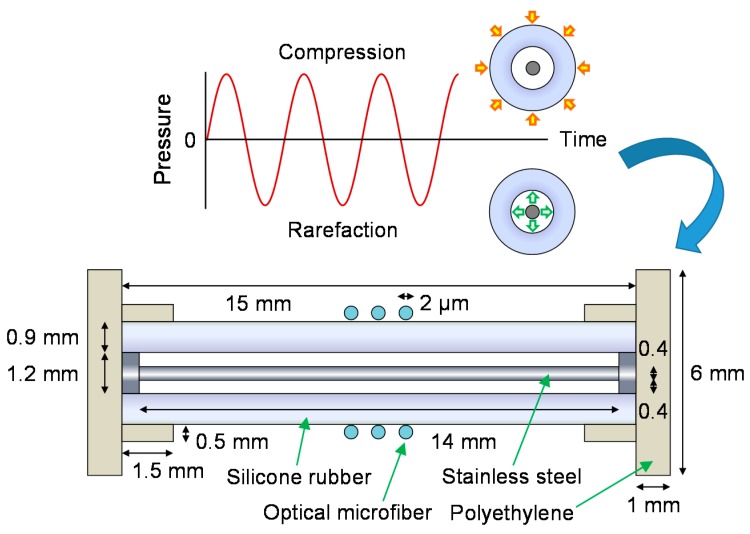
Working principle of the air-backed mandrel bound with a microfiber coil seen from the transverse perspective, and a schematic diagram of the construction seen from the axial perspective.

Chen et al. [59] introduced a compact air-backed mandrel shown in Figure 17 exploiting the excellent mechanical properties of OM. Bend radii of a few millimeters can be readily achieved with relatively low bend-loss, due to the bend insensitivity of OMs. Furthermore, such small diameters can substantially reduce the effective stiffness of the mandrel for a higher sensitivity. The air-backed mandrel operates by translating air pressure variations into bending of the mandrel walls, which then deforms the attached OM to produce a change in the OPL and thus differential phase.

**Figure 18:**
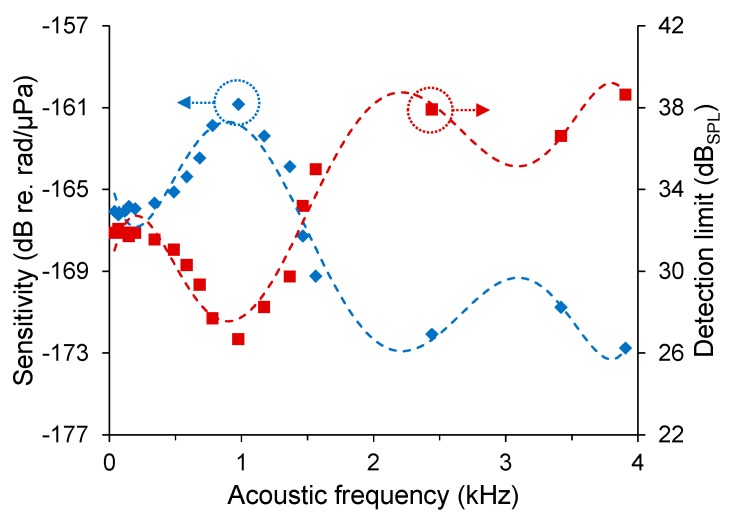
Measured frequency-resolved and sensitivities and detection limits, with polynomial fits.

An experimental demonstration was made using an MC of 2 μm core diameter and 35 mm uncoiled length, with a mandrel of 3 mm diameter and 15 mm height. The MC was embedded in the outer layer of the air-backed mandrel via UV-curable polymer. The sensitivity is 4.9 ± 0.5 mrad/Pa for an acoustic signal of 70 Hz. Its frequency response is shown in Figure 18 with average values of −165.9 dB re. rad/μPa (i.e., −136.7 dB re. rad/(μPa·m)) and –171.2 dB re. rad/μPa (i.e., −142.1 dB re. rad/(μPa·m)) from 40 Hz to 500 Hz and 1.5 kHz to 4 kHz respectively. The detection limits are also plotted as a function of frequency, with average values of 31.7 dB_SPL_ and 37.0 dB_SPL_ from 40 Hz to 500 Hz and 1.5 kHz to 4 kHz, respectively.

To increase the sensitivity, a longer OM and shorter wavelength must be used. The limiting factor is the maximum length of OM that can be fabricated. A mandrel of lower Young’s modulus and lower Poisson ratio would also be preferable. Moreover, the wall thickness of the mandrel and polymer packaging must be reduced to lower the stiffness while still maintaining clearance of the central support. The fundamental frequency and thus increase the detection bandwidth can be increased by employing a mandrel material of higher Young’s modulus, lower density and physically shorter. Since there is a conflicting requirement of Young’s modulus, there is a trade-off between sensitivity and detection bandwidth. Optimization of the mandrel parameter will deliver a good balance of the sensor parameters. To reduce the attenuation, air bubbles must be removed from the polymer packaging. The ultimate compactness of the sensor head is restricted by the diameter of the mandrel, which has to be wider than the diameter of the support pin. It is then followed by the minimum bend radius of the OM. 

## 8. Humidity Sensing

The measurement and control of humidity is useful for health, quality control and scientific purposes. Its applications range from the atmosphere, agricultural (e.g., greenhouses, crop fields), buildings (e.g., museums, heritage buildings, operating theatres, rehabilitation wards), food (e.g., baking, drying, storage), medical (e.g., smart bandages, diagnostic tools) and manufacturing (e.g., glasses, coatings, fibers). Electrical hygrometers based on resistivity or capacitance changes are well known, but they do not meet all the requirements of compact and lightweight for good portability, highly flexible to probe confined spaces, immune to EMI, completely inert within combustive environments, and sufficiently robust to last several years. Motivated by these needs, optical hygrometers [60,61,62] have been developed, which typically feature response times ranging from the order of tens of milliseconds to seconds. Sensitivites as high as 0.09 dB/%RH have been reported by Corres et al. [60] and 3.05 nm/%RH by Chen et al. [61]. The response time of current hygrometers need to be minimized for applications that can benefit from it, including: (a) process control for mass production of chemical compounds, where humidity is controlled for quality control; (b) touchless keypads for electronic systems, where response only to bare skin leads to fewer false triggers than capacitive/resistive-touch technologies; (c) water-hazard safety mechanisms for portable electronics (e.g., laptop computers) and undersea environments (e.g., the Eurotunnel); (d) respiratory analyzers for early warning of illnesses, where water vapor is less significant as noise compared to air flow; and (e) atmosphere mapping for better weather forecasts, where high spatial-resolution mapping of the atmospheric relative humidity (RH) via aerial vehicles can benefit weather simulators. Developing faster hygrometers can: (i) increase productivity; (ii) improve the safety of underwater personnel; and (iii) acquire information that can potentially benefit health. The best response times reported in the past have been 30 ms by Gu et al. (i.e., optical, nanowire) [63] and 8 ms by Mogera et al. (i.e., electrical, nanofiber) [64]. Although the latter is considerably faster, the sensor head is not resilient to EMI.

**Figure 19:**
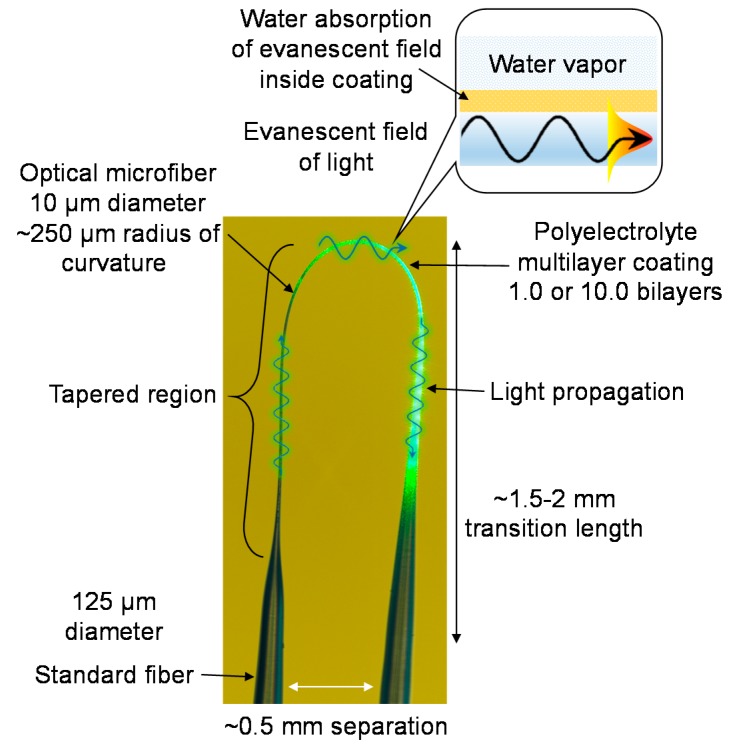
Image of OM-based hygrometer sensor head.

The fastest-ever hygrometer was recently reported by Chen et al. [65]. The hygrometer comprising a polyelectrolyte multilayer-coated OM operates by absorbing moisture from the air and attenuate the portion of light propagating through its swollen coating. The compact fiber-optic probe shown in Figure 19 has extremely short response and recovery times. Fast temporal characteristics arise from the combination of hydrophilic coating, thin coating and circular geometry. The probe is flexible and compact, which lends itself to applications involving space-limited environments.

**Figure 20:**
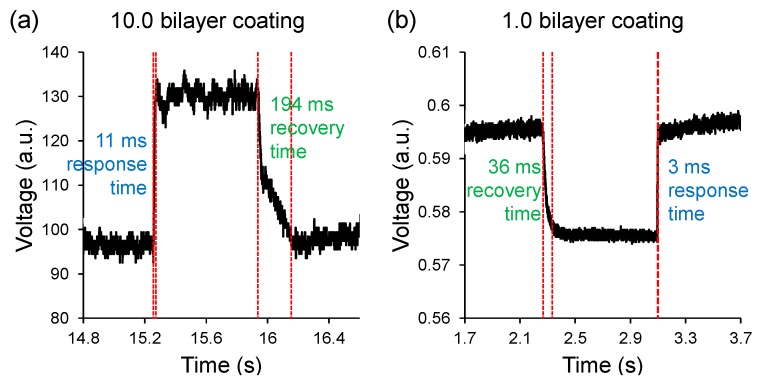
Temporal characteristics of (**a**) 10.0 bilayer coated sensor head; and (**b**) 1.0 bilayer coated sensor head.

The tested sensor heads consist of an OM of 10 µm diameter and 2 mm length configured to form a U-shaped probe, and packaged with UV-curable polymer. Each probe was functionalized with a polyelectrolyte multilayer coating of different thickness. For the 10.0 bilayer OM, the sensitivity was measured to be 0.2 %/%RH (i.e., 0–60 %RH, 80–99 %RH), and 2.7 %/%RH (i.e., 60–80 %RH). For the 1.0 bilayer OM, the sensitivity is 0.02 %/%RH (i.e., 0–72 %RH), 0.4 %/%RH (i.e., 72–80 %RH), and 0.04 %/%RH (i.e., 80–99 %RH). For the 10.0 bilayer OM, the detection limits are 3.3 %RH (i.e., 0–60 %RH, 80–99 %RH) and 0.2 %RH (i.e., 60–80 %RH). For the 1.0 bilayer OM, the detection limits were measured to be 32.6 %RH (i.e., 0–72 %RH), 1.6 %RH (i.e., 72–80 %RH) and 16.3 %RH (i.e., 80–99 %RH). For the 10.0 bilayer OM, the response and recovery times shown in Figure 20 were measured to be 11–12 ms and 175–194 ms, respectively. For the 1.0 bilayer OM, the response and recovery times were measured to be 3 ms and 36–37 ms, respectively.

There is negligible cross-sensitivity to ethanol vapor, and the selectivity to water will be exhaustively tested in the near future, in order to produce hygrometers that do not yield false results due to the presence of alcohol or other chemical vapors.

## 9. Ultraviolet-Light Sensing

Ultraviolet (UV) sensors are used in a wide range of applications, including fire detection, industrial manufacturing, biochemical research, light sources, environmental and structural health monitoring. Traditional electrical UV light sensors use photodiodes as the sensor heads, which are susceptible to EMI. To overcome this hurdle, research has been carried out on fiber-optic UV sensors based on fluorescence by Fitzpatrick et al. [66] and Joža et al. [67]; active fibers by Zmojda et al. [68] and Kim et al. [69]; and graphene oxide by Lesiak et al. [70]. They typically employ functional coatings such as phosphor that emit luminescent light in the visible spectrum under UV radiation, which is subsequently detected by a low-cost photodiode. Their advantages are immunity to EMI and high damage-threshold, which allows such UV sensors to operate in the vicinity of power lines and factories. Their sensitivities need to be improved for applications that involve detecting weak UV-light signals, such as the initial stage of wild or electrical fires. With higher sensitivity, emergency services can be alerted sooner to prevent the fire from growing too large and becoming uncontrollable.

**Figure 21:**
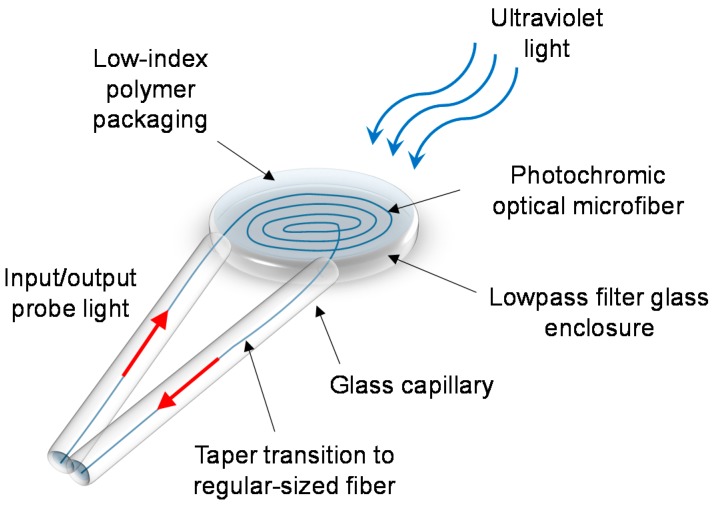
Schematic diagram of the ultraviolet-light sensor head.

**Figure 22:**
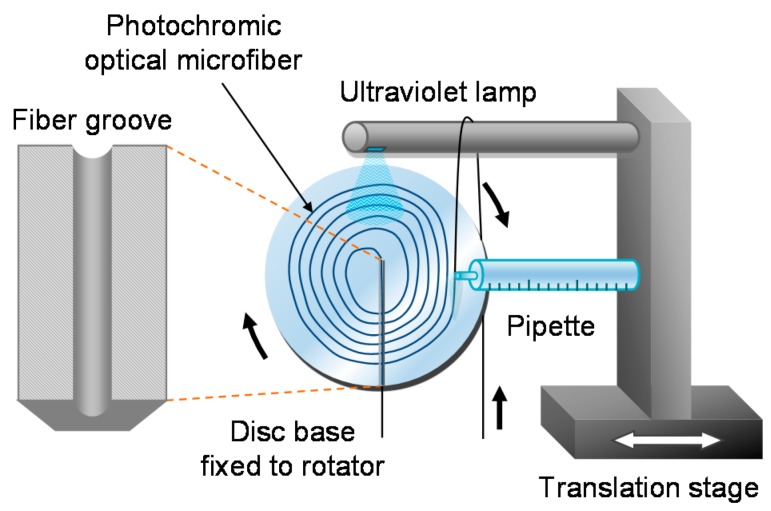
Schematic diagram of the coiling rig used during the construction of the sensor head.

In the work of Chen et al. [71], a new type of UV-light sensor based on photochromic OM was conceptually demonstrated, as shown in Figure 21. A densely packed planar coil of ZBLAN optical microfiber can be doped with a photochromic dye, and assembled in a manner shown in Figure 22. Under UV radiation, the photochromic OM experiences temporary photodarkening, and the change in the transmission of the probe light provides a measure of the incident UV light. It has the advantages of lower detection-limit than other reported sensors (e.g., 3.13 nW/cm^2^ of this work compared with 8.5 µW/cm^2^ [69]), and higher compactness if the other sensors were up-scaled in size to match its sensitivity.

For a disc of 2 cm radius packed with OM of 1 μm diameter, the predicted sensitivity and detection limit are −1.39 × 10^6^ dB/(W/cm^2^) and 3.13 nW/cm^2^ respectively. The maximum ultraviolet intensity is 22.66 μW/cm^2^. The anticipated detection bandwidth and response time are ~10 Hz and ~10 s respectively. Considering the high sensitivity, it would be of interest to experimentally demonstrate this, but it is a challenge to achieve such long lengths of OM.

To increase the sensitivity, a higher doping-concentration must be applied, and a longer length of OM must be used. Again, the upper limit depends on the maximum length of OM that can be manufactured.

## 10. Discussion

In general, in comparison to most regular-sized optical fibers, OMs can offer larger evanescent fields, stronger bend insensitivity, lower stiffness and higher configurability. The latter three attributes are unique to OMs. In effect, they can grant higher sensitivity, wider detection-bandwidth, higher compactness and lighter weight. 

As an alternative technology, small-core microstructured fibers such as the ECF exhibits the first two properties above. They can have core diameters as small as 2–3 μm [2], while the cladding diameters are in the order of ~100 μm. On the other hand, OMs can have core diameters as small as 1–2 μm [7], while the cladding diameters are irrelevant as they can be either air-clad or thinly coated (e.g., few microns). Hence, OMs can offer: (a) stronger bend insensitivity, because even though ECFs can achieve comparable bend loss to OMs when the diameter and numerical aperture of their cores are matched (i.e., same V-number), the bulkier outer-cladding of ECFs induces greater compression/tensile stresses that could possibly break first when decreasing the bend radius [3]; (b) lower stiffness resulting from the smaller cross-sectional area; and (c) higher configurability due to the possibility of forming micro-resonators. However, ECFs are more mechanically stable and robust, due to their thick outer cladding.

For sensors responding to a magnetic, electrical or thermal stimulus, a wider detection-bandwidth hosted by a shorter OPL due to the stronger bend insensitivity of MCs enable the measurement of faster-changing measurand signals. For sensors responding to a mechanical stimulus, a lower detection-limit associated with a higher sensitivity due to the lower stiffness of OMs grant an enhancement in the detection of weak measurand signals.

The size of OM-based sensor heads cannot match their ever-shrinking traditional electrical counterparts, because photons experience bend-induced loss while electrons do not. Nevertheless, compared with their conventional regular-sized fiber-optic counterparts, the many benefits of higher compactness due to the bend insensitivity of OMs include easier positioning and lower intrusiveness, which makes deployment much more practical. By significantly reducing the size of sensor heads and detection systems, miniaturization offers the possibility of portable micro-systems that can carry out many of the operations traditionally performed in a laboratory. The consequential reduction in weight leads to viable sensors for lab-on-a-chip and aerospace applications.

To compete with conventional regular-sized fiber-optic counterparts in terms of sensitivity, some OM-based sensor heads require their tapered uniform waist region to be in excess of ~10 cm, which is challenging and expensive to manufacture in high volumes. Generally, the sensitivity of non-resonator OM-based sensor heads scales with the OM length. However, the detection bandwidth associated with certain sensing mechanisms such as the Faraday effect decreases with longer OPLs. Hence, a trade-off must be considered for a good balance between sensitivity and detection bandwidth. 

OMs and its derivatives such as MCRs are notoriously difficult to handle, due to their fragile nature and sensitivity to contamination on its surface. Therefore, a cleanroom environment and automated manufacturing rigs are necessary to manufacture them with high quality and good consistency. In comparison, microstructured fibers are far more robust and can be handled in a similar fashion to single-mode telecoms fibers.

The attenuation of OMs is one of the key areas that need improvement, as they are many orders of magnitude higher than that of commercial optical fibers (~0.2 dB/km [72]). Although the theoretical minimum attenuation when embedded in UV-curable polymers is very low [73], in practice it is usually not the case. Firstly, micro-bubbles in the polymer resin cause scattering of the evanescent field [74], and contaminations of the polymer material introduce unwanted absorption. To reach near-theoretical levels of loss, a significant amount of work needs to be done to remove micro-bubbles from the polymer resin (e.g., vacuum pump) before UV-curing and to minimize contaminations (e.g., cleanroom conditions). Secondly, the wavelength of light must be optimized. Generally, the visible wavelengths range (e.g., <700 nm) will result in increased Rayleigh and Mie scattering loss in polymer resin. Certain bands must be avoided in the near-infrared wavelengths range (e.g., 700 to 1400 nm) due to the water absorption loss in polymer resin, namely 950 nm, 1244 nm and 1383 nm. The infrared wavelengths range (e.g., >1400 nm) has problems with infrared absorption loss in polymer resin. Although there are several wavelength bands that are low loss, the required light source and components are infeasible or have high costs. Hence, it is preferred to use telecom equipment at ~1550 nm that are widely available at low costs.

Another practical issue with OM-based sensor heads is the dynamic temperature range, which is determined by the polymer material used for packaging. For the Efiron PC-373 AP, the polymer experiences significant evaporation in OH content at ~80 °C and noticeable deformation by ~120 °C. Apart from the physical deterioration of the material, thermal expansion and contraction can induce internal stresses in the embedded OM, resulting in unpredictable loss and birefringence.

The lifespan of each fabricated sensor head primarily depends on the quality, age and curing state of the polymer resin. The UV-curing duration of the polymer makes a profound impact on the long-term geometrical stability and thus lifespan of the packaged sensor head. A short duration tends to avoid the build-up of internal stresses that can modify the intended OM geometry and introduce linear birefringence. On the other hand, the protective coating is more volatile and the geometrical stability of the embedded OM is vulnerable from external perturbations. Hence, it is to be appreciated that ensuring long-term reliability is still an unsolved problem. On the other hand, certain applications such as explosion and crash tests do not require the sensors involved to exhibit long lifespans, as they are expected to be damaged or destroyed after a single use. From this perspective, some applications can benefit from OM-based sensors despite their lifespan limitations, as long as the reproducibility of sensor heads is adequate.

## 11. Conclusions

We have reviewed recent advances in optical microfiber technology for the measurement of current, temperature, acceleration, acoustic, humidity and ultraviolet light. Although there are challenges for making such devices practical, they show significant potential for becoming high-performing sensors. Some of the main advantages include lower stiffness for higher sensitivity, and bend insensitivity for smaller devices. The notable limitations include high loss after packaging, and the difficulty of handle such fragile and easily contaminated strands of OMs.
